# Perceptions of Youth and Parent Decision-Making Roles Regarding Recombinant Human Growth Hormone Treatment

**DOI:** 10.3390/endocrines3040050

**Published:** 2022-10-06

**Authors:** Ettya R. Fremont, Elizabeth A. Friedrich, Chris Feudtner, Adda Grimberg, Victoria A. Miller

**Affiliations:** 1 Division of Adolescent Medicine, Children’s Hospital of Philadelphia; 2 Department of Pediatrics, Perelman School of Medicine at the University of Pennsylvania; 3 Division of Endocrinology and Diabetes, Children’s Hospital of Philadelphia

**Keywords:** short stature, growth hormone, growth hormone deficiency, decision-making, youth

## Abstract

Recombinant human growth hormone (rhGH) is prescribed to youth with growth hormone deficiency (GHD) to support normal growth and ensure healthy physical development, and to youth without GHD to address height concerns. Perceptions of youth involvement in rhGH treatment decisions have not been explored. This study aimed to examine perceptions of youth and parent roles in decisions around rhGH treatment. Youth (n = 22, 11.5 ± 2.0 years) who had undergone evaluation for short stature and their parents (n = 22) participated in semi-structured interviews after stimulation test results had been received. Interviews revealed the following themes: 1) parent provided youth with support; 2) parent facilitated youth’s decision-making involvement; 3) youth had no role or did not remember their role; and 4) youth did not remember conversations with their parents or providers. Parents facilitated their children’s involvement by sharing information and seeking their opinions. Whereas some participants described youth as having a substantial decision-making role, not all youth felt they were involved, and some youth could not recall conversations about rhGH. Parents can bolster youth involvement by having conversations using developmentally appropriate language, which is critical to youth feeling empowered and developing efficacy over their own care.

## 1. Introduction

Youth decision-making involvement (DMI) is a concept that encompasses the myriad of ways that youth contribute to decision-making processes around their medical care.^1,2^ DMI recognizes that youth have increasing desires for autonomy as they mature; however, they may not be developmentally ready to have full authority over medical decisions. For example, caregivers and providers can bolster youth involvement by soliciting their opinions about treatment, encouraging youth to ask questions,^1^ or giving them efficacy over smaller decisions (e.g., choice of injection site). Accordingly, DMI underscores the ways in which parents and providers can support youth as they develop confidence and competence in decision-making, ultimately paving the way for youth empowerment and greater understanding of healthcare across time.^1^ Research shows that youth involvement in medical decisions may lead to better treatment adherence, satisfaction, and self-efficacy,^1^ as well as greater feelings of fairness.^3^ Whereas youth experiences of treatment decisions have been explored in youth with chronic illness,^1,4–6^ to our knowledge, no research has explored youth perspectives on their involvement in the decision-making process for treatment with recombinant human growth hormone (rhGH). Pediatric rhGH treatment involves years of daily injections with a highly subjective benefit-risk balance.

Treatment for youth with growth hormone deficiency (GHD) can normalize growth and positively impact height and physical health through increases in bone mineral density, lean mass, and favorable lipid profiles,^7^ and individuals with persistent GHD may need rhGH replacement throughout adulthood when there are no height gains to be had.^8^ In contrast, children with idiopathic short stature (ISS) and other conditions that impair growth without GHD may also be prescribed rhGH to augment growth.^9^ However, height increases from rhGH treatment for youth with ISS are more modest and less consistent than for youth with GHD, with some undergoing daily injections for years to experience no height increase. Youth with ISS who take rhGH experience an average height gain of 1.9 inches,^10^ which is below the 4 inches that a majority of parents of patients at a subspecialty growth center indicated would be necessary to mitigate quality of life concerns for their child.^11^ All this must be balanced against the logistical and emotional burdens on youth (e.g., frustration with injection frequency, bruising) and parents (e.g., feelings of guilt, interference with daily life).^12^ Furthermore, treatment with rhGH may result in unwanted side effects, including slipped capital femoral epiphysis that requires surgical pinning and intracranial hypertension in the short term, and unknown effects in the long term.^7,13^

Given the burdens (e.g., daily injections) associated with rhGH treatment, and the benefits of youth DMI (e.g., treatment satisfaction, better adherence), youth involvement in the decision to pursue rhGH may be beneficial, especially when it is elective, i.e., prescribed solely for increased height. Understanding how youth and parents view youths’ roles in decision-making may pave the way for developing strategies to facilitate greater youth involvement and enhance self-efficacy around rhGH usage. Therefore, the goal of the present study was to explore and describe parent and youth perceptions of youth roles in the decision-making process for rhGH treatment.

## 2. Materials and Methods

### 2.1. Research Design

Data in the present study were collected from youth and their caregivers (hereafter referred to as parents) who were recruited as part of a broader longitudinal, mixed-method, multi-reporter study exploring quality of life and psychosocial adjustment in youth receiving subspecialty care for short stature. Study participants filled out questionnaires over approximately two years and six visits. A subset of randomly selected participants also completed semi-structured interviews at visits 1, 2, and 6. Visit 2 interviews, from which data in the present manuscript were drawn, were conducted after youth participants received results from their GH stimulation testing and outcome of their overall evaluation for possible GHD.

### 2.2. Recruitment and Enrollment

Youth ages 8–14 years were identified by weekly reports of patients scheduled for GH stimulation testing at the Children’s Hospital of Philadelphia (CHOP) Day Medicine Unit. Youth were excluded if they had developmental delay, a physical disability requiring accommodations for daily living, a life-threatening medical condition, or a past psychiatric hospitalization (due to the potential impact of the history of serious mental health concerns on quality of life). All participants were English-speaking. One parent per family was eligible to participate. Recruitment for this prospective observational study began in May 2019, and data in the present study were from participants recruited prior to January 2022. Chart review identified 244 potentially eligible parent-youth dyads to whom letters were sent. 177 families were reached and informed about the study by phone, with 111 parents expressing interest in participation. Of those who expressed interest, 72 parent-youth dyads were reached upon callback for screening and enrollment, of these seven were deemed ineligible and three youth declined. Ultimately, 62 dyads enrolled in the study. Consent and verbal permission/assent occurred during screening.

Using computer generated random numbers, researchers selected 26 dyads to complete semi-structured interviews at Visits 1, 2, and 6. Twenty-six dyads would ensure reaching thematic saturation, or the number of interviews required such that no new themes would be expected to emerge from the data,^14^ while providing a buffer for attrition across time. The timing of Visit 2 interviews was anchored to and occurred within 3 months after the GH stimulation test. By Visit 2, families had received their GH stimulation test results, but may not have met with their providers to review results. There were no demographic differences between dyads selected for interviews and dyads in the questionnaire-only group (all p > 0.10). Prior to Visit 2, three dyads (11.5%) were withdrawn from the interview group due to ineligibility or attrition. Two additional participants (one parent, and one youth) were excluded from Visit 2 analyses due to poor quality audio-recording. Ultimately, 23 dyads were represented in the Visit 2 interviews, above the number typically required for thematic saturation.^14,15^

### 2.3. Procedures

The study was approved by the institutional review board of the Children’s Hospital of Philadelphia. Visit 2 semi-structured interviews were conducted separately and via telephone for youth and parents.

Two Masters level research assistants (RAs) were trained by the principal investigators (VAM and AG) to conduct semi-structured interviews. Interviews were audio-recorded, transcribed, and uploaded into NVivo 11 software (QSR International, Melbourne, AU) for analysis. Participants were compensated for their time with MasterCard gift cards.

### 2.4. Measures

Parents completed a demographics questionnaire for themselves and their child at Visit 1. Data included sex, gender, age, and race/ethnicity. Other parent information included highest educational grade completed, annual household income stratum, employment status, and marital status.

Visit 2 semi-structured interview questions focused on participants’ perspectives about decision making related to rhGH. Participants were asked broad-based questions, including “Tell me more about the decision about starting growth hormone. Will you be starting rhGH?” If they responded affirmatively, they were asked, “Why?” If not, they were asked, “Why not?” Participants were also asked about parent, child, and physician roles in the decision-making process (e.g., “Overall, how would you describe your role in the decision about growth hormone treatment?”). Other questions were more targeted, but still open-ended. For example, youth (and parents) were asked, “Did you and your parents (child) agree or disagree about starting/not starting growth hormone?” Prompts were used throughout the interviews to encourage participants to elaborate.

Between Visits 1 and 2, research personnel completed a medical record review to document mid-parental height, serial height data [including absolute measurements and age and gender-specific standard deviation scores (SDS)], Tanner stage, weight and BMI SDS, and bone age SDS. Peak GH concentration on stimulation testing was collected.

### 2.5. Coding and Analysis

Semi-structured interviews were coded using NVivo and were analyzed using a modified inductive approach.^16^ First, to generate an initial coding scheme, two RAs separately read through a selection of interview transcripts from Visit 1 (eight parents and 10 youth) and Visit 2 (four parents and four youth). AG and VAM then read the transcripts, reviewed the RAs’ coding schemes, and integrated the schemes into a single coding structure based on team discussion. The two RAs then coded the transcripts using this coding structure. 20% of participants’ transcripts were double coded to ensure dependability of the data.

After transcripts were coded according to the initial coding scheme, the second step of the analysis process was to identify themes relevant to participants’ perceptions of the decision-making process. The first author (ERF) developed summaries of participants’ interviews, which were then grouped by parental report of rhGH treatment status, i.e., whether the youth started or would be starting rhGH, or would not start rhGH. Next, based on the summaries, ERF identified key themes that had emerged from the data to determine overlapping and distinct themes based on treatment status. Final themes were agreed upon through an iterative process involving discussions with and review by the senior author (VAM).

## 3. Results

### 3.1. Participants

Visit 2 interviews were completed by 22 youth and 22 parents representing 23 dyads. Comprehensive baseline (i.e., Visit 1) interviewee characteristics are presented in Table 1. Six parents reported that their child would be starting rhGH treatment, and 17 parents reported that they were either still in the process of deciding or would not be starting rhGH as of Visit 2. Due to the timing of interviews, some final decisions about starting rhGH treatment had not yet been made.

### 3.2. Overview

Four overarching themes, including five subthemes, emerged from analysis of parent and youth interviews about youths’ roles in the rhGH decision-making process (TABLE 2). Overarching themes included: 1) parent provided youth with support; 2) parent facilitated youth’s decision-making involvement; 3) youth had no role or did not remember their role; and 4) youth did not remember conversations with their parents or providers. Unless otherwise specified, themes emerged regardless of whether dyads had started rhGH treatment. Table 2 contains a comprehensive overview of themes, subthemes, and representative quotes.

### 3.3. Theme 1: Parent Provided Youth with Support

Both parents and youth reported that parents had supported youth emotionally and logistically throughout the decision-making process.

#### 3.3.1. Emotional support

Participants explained that parents provided emotional support to youth. Several youth mentioned that parents’ emotional support largely centered around assuaging their concerns about needles and daily injections. A few parents noted that their emotional support would extend to helping youth cope with any side effects of treatment or potential treatment.

#### 3.3.2. Logistical support

Participants explained that parents provided logistical support to youth in a variety of ways throughout the decision-making process. For example, participants mentioned that parents monitored their child’s growth, researched rhGH and its side effects, scheduled and brought their child to appointments, talked with their child’s provider, and purchased and administered rhGH.

### 3.4. Theme 2: Parent Faciliated Youth’s Decision-Making Invovlement

Parents facilitated youth’s involvement in the decision-making process by 1) sharing information with their child, although some parents limited the information that was shared; 2) seeking youths’ thoughts and opinions about rhGH treatment; and 3) facilitating a mutual or substantial decision-making role for youth.

#### 3.4.1. Parent shared information with their child

Several participants mentioned that parents shared information throughout the decision-making process. For example, some parents talked with their child about the results of the stimulation test. Parents explained the purpose of rhGH (i.e., facilitate growth) and gave information about the treatment process (e.g., treatment duration, injection frequency).

Some parents discussed intentionally limiting the information they gave to their child. Parents hesitated to fully disclose information that could be uncomfortable or anxiety provoking (e.g., daily injections). For example, one mother whose 13-year-old son (#021) was starting rhGH explained that his brother had experienced complications (i.e., eye tumor) while taking rhGH. Although she did not hide that information from her son, she stated that she “want[ed] him to kind of recognize the symptoms...but we didn’t go into great detail.” Another mother and her spouse gave their 11-year-old daughter (#007) just enough information so that their daughter “wasn’t curious as to why she was going for this test...” She elaborated that they would have had a broader discussion with their daughter if they were at “that point where we had to...decide.”

#### 3.4.2. Parent sought their child’s opinions and thoughts about treatment

Participants stated that parents asked for their child’s thoughts and opinions about rhGH treatment. For example, several parents asked their child directly whether he or she wanted to pursue treatment. Some parents also engaged their child in discussions about rhGH. One youth described how his parents encouraged him to converse with his endocrinologist by helping him figure out what questions to ask.

#### 3.4.3. Youth had a mutual or substantial decision-making role

Several participants stated that the youth’s role in the decision-making process was as large or larger than their parents’. For example, one mother (#058) in a dyad that had decided to pursue treatment said that she was a “joint decision-maker” with her daughter (age 10), and that as a parent, her role was “just [to be] on board with what she [my daughter] wanted.” Relatedly, participants who were not starting rhGH treatment indicated that if, in the future, they were confronted with the option of pursuing treatment, the youth would have an equal or greater role than the parents.

Several participants emphasized that the child’s agreement was necessary to pursue rhGH treatment (or would be necessary to begin rhGH treatment for dyads who were not presently starting rhGH). Parents emphasized that their child’s agreement was necessary to begin treatment because of bodily autonomy (i.e., the burden of daily injections falls on the child). Parents explained that if their child were hesitant or did not want rhGH, they would not pursue treatment. Relatedly, some youth were aware that their parents would seek their approval to begin treatment.

### 3.5. Theme 3: Youth Had No Role or Did Not Remember Their Role

This theme emerged only for dyads who were not starting rhGH presently. Some youth stated that they did not have a role or did not remember their role in the decision-making process, with a few stating that decision-making was up to their parents and provider. Relatedly, some parents expressed that because they were told by their child’s endocrinologist that their child did not qualify for rhGH, neither they nor their child had a role in the process.

### 3.6 Theme 4: Youth Did Not Remember Conversations with Their Parents or Providers

Some youth stated that they did not remember having conversations about rhGH, including conversations about the results of their stimulation test, with either their parents or providers. Other youth remembered having a conversation but did not remember the content of that conversation. As one youth (age 8, #004) explained, she and her parents “sort of” talked about rhGH, but she did “not really” remember what was discussed. Additionally, some youth and their parents had differing perspectives of whether a conversation took place. For example, when asked about whether she’d heard about her testing results or next steps, one youth (age 11, #029) said “Uhh. No, I don’t remember.” However, her mother explained that she and her daughter’s endocrinologist both spoke to her about next steps: “So, I – yeah, I talked to her about it. She was able to understand, also [name of endocrinologist] himself spoke to her and he was very clear...you know...she’s only 11 but...I think she understands.”

## 4. Discussion

In the present study, parents and youth described ways that some parents supported and facilitated their child’s involvement in the decision-making process around rhGH treatment. Parents provided emotional and logistical support, gave their children information about rhGH, and sought their child’s thoughts and opinions about the treatment. In other medical decision making contexts, including youth in decisions about their treatment may help youth feel empowered and may decrease anxiety about treatment;^17^ not doing so may lead them to feel powerless, angry, or disappointed.^18^

Youth in the present sample played a substantial role in the decision-making process about rhGH, which is consistent with studies of youth DMI for treatment of chronic diseases such as type 1 diabetes.^4,19^ Previous research on youth DMI in medical settings indicated that youth desire a large role in the decision-making processes surrounding their own care^19^ in conjunction with support from parents and providers.^4,19,20^ In the present study, parents supported youth DMI in a variety of ways, including asking their child questions and providing information. Autonomy supportive communication in youth with chronic disease has been linked to multiple benefits, including treatment adherence.^21^ Recognizing youth autonomy may lay the foundation for youth to gain self-efficacy in decision making and become competent in their own care.^22^ Parental autonomy support may enhance youth’s ability to make decisions aligned with their personal needs, rather than external pressure.^23^ In the more immediate term, youth efficacy over decisions regarding their treatment may lead to better adherence to treatment regimens,^6^ which may be especially important given suboptimal adherence rates in pediatric rhGH treatment.^24^

Participants in this study reported that parents provided both emotional and logistical support, which is consistent with findings of other studies of parent and youth decision-making roles in healthcare.^4^ Youth in the present study stressed that parents’ emotional support around the daily injections helped quell their concerns about the size of needles and how painful the injections would be. Such validation and normalization of children’s concerns may reduce anxiety in medical settings, thus allowing youth to feel comfortable and safe expressing themselves.^25^

Another way in which parents in the present sample supported their child’s DMI is by providing information; in turn, youth relied on their parents to provide information about rhGH. Previous literature suggests that sharing information with youth in a developmentally appropriate way is critical to helping youth become involved in their own care.^26,27^ Whereas withholding certain information may restrict youth’s involvement,^27^ there may be some instances where filtering or limiting information may help facilitate youth DMI. For instance, heightened emotional states, such as feeling sad, could lead youth to disengage from the decision-making process.^26^ A few parents in the present sample indicated that they limited giving information to their children that may have increased their child’s anxiety (e.g., information about route and frequency of rhGH administration).

Despite the ways in which parents facilitated youth involvement throughout the rhGH decision-making process, youth involvement was not consistently reported across all youth. One barrier to youth involvement included youth reporting either not having conversations or not remembering specifics of conversations about rhGH with either their providers or their parents. Iterative conversations would provide an opportunity for parents to ease youth into the more disquieting aspects of treatment (e.g., the daily injections) without overwhelming youth. Previous research indicates that youth decisions about treatment may occur over the course of several conversations.^4^ Finally, youth may have varying preferences about their degree of involvement,^20^ which parents and providers should take into consideration.

### 4.1. Clinical Implications

Whereas youth who are undergoing evaluation for short stature may not have the cognitive maturity to make the final decision over treatment, results from this study provide insight into the ways in which parents and providers can bolster youth involvement throughout the evaluation and management of short stature. Importantly, parents may need to have several conversations with their children about rhGH treatment. In addition to information about the purpose of the treatment, parents and providers should also consider giving youth information about treatment duration and what treatment entails (i.e., daily injections). Relatedly, providers should ensure that parents and youth understand the risks and benefits of undergoing or abstaining from rhGH treatment for GHD or ISS. Given some youths’ anxiety and hesitancy around the injection part of treatment, parents and providers should be sensitive and attentive to youth’s individual information needs. For example, for some youth, having several conversations may be beneficial, easing them into the idea of daily injections. Parents and providers may also consider providing information in a variety of ways. For example, in a study regarding continuous glucose monitoring in youth with type 1 diabetes, participants reported that videos or demonstrations of peers using CGM devices helped alleviate their fears about the device.^4^ A demonstration may be particularly helpful as research indicates that few youth undergoing rhGH treatment report experiencing pain with the injections.^28,29^

Additionally, parents should consider having in-depth discussions with their child about the potential benefits and drawbacks of rhGH treatment. Because youth seeking rhGH treatment may still be in middle to late childhood (or even younger), parents and providers should take care to use developmentally sensitive language and ensure their child’s understanding. Iterative conversations may be critical to ensuring that youth understand why they may need treatment and what treatment entails. Strategies that facilitate discussions with youth involve seeking youth’s opinions, inquiring whether youth have any questions remaining, and asking whether youth understand what was discussed.^1^ Directly engaging with youth signals that the youth’s opinions are important, and may help increase youth efficacy in expressing themselves.^1^ Providers can also guide parents about best practices to address youth concerns or questions about treatment.^1^ By including youth in discussions and facilitating their active involvement, parents and providers can be more attuned to youths’ concerns and opinions about treatment, which may help youth feel more empowered in their own care.

### 4.2. Limitations

Results of this study should be interpreted in light of several limitations. First, as is reflective of the population undergoing GH stimulation testing in the United States, were drawn from a population of mostly White, non-Hispanic patients from higher SES households;^30^ similarly, parent participants were mostly mothers, White, and college educated, and males outnumbered females among the patients.^31^ Therefore, results may not be generalized to fathers’ perceptions of their and their children’s roles in the decision-making process or to families from more diverse demographic backgrounds. Because Visit 2 semi-structured interviews occurred after youth had undergone a full evaluation (including the stimulation test) for short stature, this study was not designed to achieve thematic saturation based on youth uptake of rhGH treatment or not. Furthermore, some youth may have denied remembering or participating in conversations that may have been emotionally upsetting (i.e., ascertainment bias); however, notably, our youth participants talked about many aspects of short stature and the impact on their lives during the interviews. Further, response bias, the tendency to want to provide the more socially desirable response, may have shaped participant comments.

### 4.3. Future Research

This study provides insight into youths’ roles in the decision-making process around rhGH treatment. However, given that rhGH treatment duration may span childhood and adolescence, longitudinally exploring youth roles and perceptions from diagnosis through treatment duration may provide insight into whether these decisions should be revisited as youth mature and their preferences for treatment and involvement change. An understanding of whether and how youth involvement changes may provide insight into how parents and providers can support youth’s confidence in decision-making involvement from the outset, thus setting the stage for engagement across time.^1,32^ Future quantitative analyses from the broader study sample will explore the relation between youth DMI and satisfaction with rhGH treatment and trajectories of youth QoL and self-esteem after GH stimulation testing for youth diagnosed with GHD, as well as for youth with ISS both treated and not treated with rhGH. Additionally, future research may help elucidate whether and how DMI in GH treatment is associated with increased adherence to rhGH treatment. Finally, whereas sample characteristics were reflective of families seeking subspecialty care for short stature in the United States, future research should explore DMI in more diverse samples.

## Figures and Tables

**Table 1.** Demographic and Patient Characteristics for Participants who Completed the Baseline Interview (n = 24)

Variable	n (%) or mean + SD [range]

Youth age (years)	11.5 + 2.0 [8, 14]
Youth sex (female)	6 (25.0)
Youth race	
White	20 (83.3)
African-American	1 (4.2)
Asian	1 (4.2)
Other	1 (4.2)
Multi-racial	1 (4.2)
Youth Hispanic ethnicity (yes)	2 (8.3)
Parent sex (female)	22 (91.7)
Annual household Income (USD)	
< 20,000–39,999	1 (4.2)
40,000–59,999	0 (0.0)
60,000–79,999	3 (12.5)
80,000–99,999	3 (12.5)
More than 100,000	15 (62.5)
Refused	2 (8.3)
Parent education	
Some or completed high school	1 (4.2)
Some college or technical school after high school	5 (20.8)
College graduate	3 (12.5)
Some post-college graduate education	2 (8.3)
Masters, PhD., MD, law degree, etc.	13 (54.2)
Parent employment status	
Not currently employed	3 (12.5)
Working part-time	4 (16.7)
Working full time	17 (70.8)
Parent relationship status	
Single - Divorced	3 (12.50)
Single - Widowed	1 (4.2)
Married - First Marriage	18 (75.0)
Married - Not First Marriage	2 (8.3)
Height (cm)	134.6 + 13.4 [111.4, 156.0]
Height z-score	2.2 + 0.51 [ 3.2, 1.4]
Weight z-score	1.5 + 0.8 [ 2.7, 0.5]
BMI z-score	0.2 + 0.7 [ 1.4, 1.5]
Mid-parental height (MPH) z-score	0.06 + 1.2 [ 2.7, 2.3]
Height z-score minus MPH z-score	2.2 + 1.0 [ 3.8, 0.2]
Bone age (years)	10.4 + 2.3 [6.3, 13.5]
Tanner stage	
1	13 (54.2)
2	6 (25.0)
3	3 (12.5)
Unknown	2 (8.3)
Peak GH concentration on stimulation testing (ng/ml)	
>10	9 (37.5)
≤10	15 (62.5)

**Table 2.** Themes, Subthemes, and Representative Parent and Youth Quotes

Theme/Subtheme	Parent Quote	Youth Quote

Parent Provided Youth with Support		

Emotional support	Basically, just his mom, so if he needs advice or needs to talk about things or if he’s feeling any side effects, I would be the one that he would go to...[He] can, you know, ask me any questions he wants, and I’m open, and I’m there for him so... - son age 13 (#031)	[My dad told me] that it’s probably – it’s not going to hurt. It’s like a little baby, infant – it’s a needle like you get like when you’re baby, kind of like an EpiPen needle. So it’s probably not going to affect me. And hopefully I’ll be able to participate in more stuff by the end of it. - boy, age 12 (#055)
Logistical support	I’ll be the one making all the appointments and getting him there and explaining everything to him, with any questions he has other than the doctor, because he has great questions when we go. - son age 12 (#057)	Them giving it [rhGH to] me - giving me the shots and caring enough about me...them buying it for me - boy, age 13 (#021)

Parent Facilitated Youth’s Decision-Making Involvement		

Shared information with their child	... I kind of try and walk this line of like giving him information but not over – like not making him nervous. And so we wanted to be upfront with why we were doing it and what he was doing. And he briefly asked like... ‘if I have to keep going, like what is that, is it like a pill, is it?’ – and so we had briefly kind of talked about, ‘well, we don’t have all the answers, but it is not a pill, it would be more, we believe, like a shot, but we don’t know all of the information, so it’s not worth getting upset about.’ But we kind of try and walk that line of giving him information but not totally terrifying him, I guess. - son age 10 (#052)	...they [my parents] were very helpful with research on the growth hormone and, uh, uh, looking for words to describe it...- boy, age 12 (#006)
Parent sought their child’s opinions and thoughts	Well I’d say my role is just to keep supporting him, making sure I’m asking him questions, making sure that he’s being seen medically and that we’re tracking it closely...–monitor[ing] our experience and, um, understanding when he’s - you know, he’s being hormonal kind of thing.- son age 13 (#021)	Well, my mom and dad have not been forcing me. They didn’t force me to get the IV. It was – they said that they had the possibility to do it earlier, and they asked me if I was okay with that. And so, yes, they’ve been asking my opinion about stuff. – boy age 10 (#052)
Youth had a mutual or substantial decision-making role	Well, it’s hard – it wasn’t – I guess it wasn’t – it was a no-brainer, I guess, was the best way to describe it. [Child Name] was interested in this as soon as it was brought up. And I guess my role was I was just on board with what she wanted and like I definitely have noticed how small she is compared to other kids in her age group. So I think my role was probably joint decision maker with [Child Name]. – daughter age 10 (#058)	So like my mom said – like my mom and my dad, they said like they are fine, like whatever I wanted. I wanted to get the shot, so like they were – like they didn’t really care if I got it or not. But like they wanted me to be happy no matter what I did. – girl, age 10 (#058)

Youth Had No Role or Did Not Remember Role	Interviewer: So do you feel like [child’s name] played a role at all?... Participant: Not really. I mean he just - you know, he - he seemed like he was happy with - with like what I had to say, you know. I mean if it - if it had - if it had gone the other way like I think he would have been a little nervous. – son age 8 (#003)	I wasnť a part of – I wasn’t a part of the decision making. Thaťs all my mom...my mom and my endocrinologist. –boy, age 11 (#061)

Youth Did Not Remember Conversations with Parents or Providers	N/A	Interviewer: Okay. Do you remember, um, hearing about what your results were from that test? Participant: Um, no, I don’t think so. – girl age 11 (#029)

## Data Availability

Not applicable.
